# Thyroid dysfunction from inhibitor of fibroblast growth factor receptor

**DOI:** 10.1186/s40164-019-0130-4

**Published:** 2019-02-18

**Authors:** Jeffrey Ahn, Justin Moyers, John Wong, Chung-Tsen Hsueh

**Affiliations:** 1 Department of Internal Medicine, Loma Linda University, Loma Linda, CA 92354 USA; 2 Division of Medical Oncology and Hematology, Department of Internal Medicine, Loma Linda University, 11175 Campus Street, CSP 11015, Loma Linda, CA 92354 USA

**Keywords:** Hypothyroidism, AZD4547, Thyroid dysfunction, Fibroblast growth factor receptor, Tyrosine kinase inhibitor, Urothelial cancer, Case report

## Abstract

**Background:**

Thyroid dysfunction has not been previously reported in clinical trials of selective fibroblast growth factor receptor (FGFR) inhibitors including AZD4547. Herein, we report a case of worsening hypothyroidism in a patient with advanced urothelial cancer treated with AZD4547.

**Case presentation:**

An 80-year-old Caucasian female with metastatic urothelial carcinoma failed first-line chemotherapy with gemcitabine and carboplatin and second-line treatment with atezolizumab, an inhibitor of programmed cell death ligand 1. She developed hypothyroidism at completion of atezolizumab treatment and responded to levothyroxine. Subsequently she was enrolled to a phase II study and received AZD4547 due to an actionable mutation at FGFR3 found in tumor biopsy. Two months later, she experienced recurrent hypothyroidism symptoms, and was hospitalized twice for small bowel obstruction. Her thyroid stimulating hormone level was significantly increased to 2957 uIU/mL (reference range 0.8–7.7 uIU/mL). Her levothyroxine dose was increased accordingly. Her thyroid function returned to normal 1 month afterwards, and small bowel obstruction did not recur.

**Conclusion:**

Further reports and studies will be needed to confirm the relationship between AZD4547 and hypothyroidism. Based on this observation and possible mechanisms for thyroid dysfunction discussed in this paper, routine thyroid function monitoring in patients receiving FGFR inhibitor should be considered.

## Background

Thyroid dysfunction, especially hypothyroidism, is frequently seen in cancer patients receiving tyrosine kinase inhibitor (TKI) or immune checkpoint inhibitor treatment [1, 2]. The incidence varies due to different mechanisms of action. Unrecognized hypothyroidism may lead to severe gastrointestinal manifestations such as decreased colonic motility, constipation and bowel obstruction [3, 4].

Fibroblast growth factor receptor (FGFR) is a transmembrane receptor tyrosine kinase family, which consists of four members (FGFR 1–4) that are involved in cell signaling by binding to fibroblast growth factors (FGFs) [5]. The downstream singling of FGFR involves mitogen-activated protein kinase (MAPK) and PI3K-AKT pathways, which are essential in cell growth and oncogenic events. Aberrations in the FGFR signaling pathway have been implicated in the development and progression of multiple solid tumor malignancies [6]. A number of novel drugs targeting the FGFR cascade have been developed and are undergoing clinical trials. AZD4547 is an orally available, small-molecule tyrosine kinase inhibitor that selectively inhibits FGFR 1–3 [7]. The drug has demonstrated anti-tumor activity in FGFR amplified gastric and non-small cell lung cancer xenograft models in mice [8, 9]. In a phase I study of patients with advanced solid malignancies, AZD4547 was found to be well tolerated without dose-limiting toxicities, and approximately 70% of patients had stable disease > 4 weeks [10].

Phase I and II clinical trials of AZD4547 revealed acceptable safety profiles with common adverse effects being gastrointestinal toxicities (nausea/vomiting, decreased appetite and bowel habit changes), fatigue, hyperphosphatemia, dry eye, epithelial and mucosal dryness, stomatitis, retinal pigment epithelial detachment, etc. [10–12]. Thyroid dysfunction has not been previously reported in trials involving FGFR inhibitors including AZD4547. Herein, we report a case of worsening hypothyroidism in a patient with advanced urothelial cancer receiving AZD4547.

## Case presentation

An 80-year-old Caucasian female was referred for left renal mass and multiple expansile bone lesions resulting in back and chest wall pain in September 2016. She had a history of in situ high-grade papillary transitional cell carcinoma of the urinary bladder, status post radical cystectomy and pelvic lymph node dissection in May 2012. She underwent left renal mass biopsy which revealed invasive urothelial carcinoma. She received first-line palliative chemotherapy with gemcitabine and carboplatin along with palliative radiation therapy for pain control. Subsequently she also received denosumab, a bone modifying agent, for symptomatic bone metastases after dental evaluation. Due to progressive disease, she started second-line systemic treatment from December 2016 with atezolizumab 1200 mg intravenously over 60 min every 3 weeks, an inhibitor of programmed cell death ligand 1 [13]. She tolerated atezolizumab well but developed progressive disease in May 2017. At the end of atezolizumab treatment course, she developed clinically significant hypothyroidism with cold intolerance, insomnia, constipation and extreme fatigue in the setting of elevated thyroid stimulating hormone (TSH) level to 21 uIU/mL (reference range 0.8–7.7 uIU/mL). Therefore, she was started on levothyroxine, and her symptoms resolved with normalization of TSH.

Afterwards she was enrolled to a phase II study (ClinicalTrials.gov Identifier: NCT02465060) and underwent tumor biopsy at sternum for next-generation sequencing assay which revealed an actionable mutation at FGFR3. She received AZD4547 80 mg orally twice a day from June 2017 on clinical trial. After 2 months of AZD4547 treatment, she experienced recurrent hypothyroidism symptoms, and was hospitalized twice for small bowel obstruction responding to medical treatment. Her TSH level at that time was significantly increased to 2957 uIU/mL, and daily levothyroxine dose was increased from 50 to 100 mcg accordingly. Her thyroid function returned to normal 1 month afterwards, and she did not experience recurrence of small bowel obstruction.

She achieved stable disease on AZD4547 for approximately 6 months prior to development of progressive disease. She progressed through subsequent lines of treatment with cisplatin and docetaxel and finally pemetrexed before succumbing to disease in June 2018.

## Discussion and conclusion

Hyperphosphatemia has been shown to be a class-specific mechanism-based toxicity of selective FGFR inhibitors including AZD4547 with incidence ranging from 30 to 65% in clinical trials [7]. The mechanism of action is likely due to interruption of FGF23 binding to FGFR on the proximal renal tubule which results in increased phosphate reabsorption and reduced renal production of vitamin D [10, 14]. The nonselective FGFR TKIs such as E3810 are multitarget TKIs that include FGFR and vascular endothelial growth factor receptors in their panel of targets [15]. Nonselective FGFR TKIs lack kinase selectivity and hypothyroidism has been shown to be a common adverse effect in this class of compounds.

Immune checkpoint inhibitors such as monoclonal antibodies against programmed death 1 and programmed cell death ligand 1 have been approved for the treatment of patients with locally advanced or metastatic urothelial carcinoma [16]. Most thyroid dysfunction induced by immune checkpoint inhibitors is hypothyroidism derived from immune-related thyroiditis [2]. It is recommended to monitor thyroid function prior to and during treatment with immune checkpoint inhibitors, and initiate hormone replacement therapy or medical management of hyperthyroidism as clinically indicated. In clinical studies of atezolizumab, hypothyroidism occurred in 4.6% of patients and hyperthyroidism occurred in 1.6% of patients [17]. Our patient developed symptomatic hypothyroidism after 5 months of atezolizumab treatment and responded to levothyroxine. Per study protocol, she did not receive thyroid function monitoring during subsequent treatment with AZD4547. Therefore, she developed worsened hypothyroidism leading to hospitalization with bowel obstruction.

One plausible mechanism for FGFR inhibitor inducing hypothyroidism is inhibiting the angiogenesis of thyroid gland vascular bed and subsequently causing hypoperfusion and local destruction of the organ. Presta et al. demonstrated that FGFR1 and FGFR2 are present on endothelial cells and postulated that FGFs exert their angiogenic mechanism via paracrine signaling by stromal cells [18]. Preclinical model of microvascular endothelial cells grown on a three-dimensional collagen gel, FGF2 were shown to induce cells via direct effect to invade the underlying matrix by forming capillary-like tubules and initiate angiogenesis [19]. In murine model, FGF1 and FGF1/FGF2 double knockout mice had poor wound healing compared to normal control mice [20]. In 2004, Davis et al. demonstrated that thyroxine has proangiogenic role in the chick chorioallantoic membrane model and its angiogenic effect is mediated by FGF2 and MAPK dependent [21].

Similar to other TKIs such as imatinib and sunitinib, cross reactivity between TKI and non-target substrates can cause side effects. A prospective study by de Groot et al. demonstrated that nine of fifteen patients with advanced medullary thyroid cancer status post thyroidectomy on levothyroxine who received imatinib had to increase thyroxine hormone requirement while on therapy. The authors hypothesized that imatinib increased thyroid hormone clearance through induction of hepatic microsomal enzymes including mixed function oxygenase and uridine diphosphate-glucuronosyltransferase [22]. Rather than affecting the thyroid gland itself, this mechanism suggests that TKI could interfere with active serum level of thyroid hormone leading to worsening of hypothyroidism similarly to what our patient experienced.

Furthermore, another possible mechanism for TKI-induced hypothyroidism is direct interference of the thyroid gland’s function by blocking iodine uptake and inducing antiperoxidase activity. In a prospective phase I–II study, Mannavola et al. reviewed thyroid function test, thyroid ultrasound as well as radioiodine uptake testing of 24 patients with gastrointestinal stromal tumor treated with sunitinib. Impaired radioiodine uptake was observed by a blunted early radioactive iodine uptake curve suggesting an alteration in the uptake process [23]. Wong et al. demonstrated the antiperoxidase activity of sunitinib by looking at the effect on guaiacol oxidation and protein iodination through lactoperoxidase and concluded that the drug had one-fourth potency of propylthiouracil suggesting potential antiperoxidase activity of TKI [24].

Further reports and studies will be needed to confirm the relationship between AZD4547 and hypothyroidism. Based on this observation and possible mechanisms for thyroid dysfunction discussed in this case, routine thyroid function assessments in patients receiving FGFR inhibitor should be considered.

## Abbreviations

TKI: tyrosine kinase inhibitor
FGFR: fibroblast growth factor receptor
TSH: thyroid stimulating hormone
FGF: fibroblast growth factor
MAPK: mitogen-activated protein kinase
